# Restraint reduction in general hospital care by preventive patient involvement: a pilot study

**DOI:** 10.1186/s12877-025-06015-3

**Published:** 2025-05-20

**Authors:** Silvia Thomann, Isabelle Barbezat, Karin Thomas, Sandra Siegrist-Dreier, Chantal Britt, Sabine Molls, Sabine Hahn, Dirk Richter, Kai-Uwe Schmitt

**Affiliations:** 1 School of Health Professions, Bern University of Applied Sciences, Applied Research & Development in Nursing, Murtenstrasse 10, Bern, 3008 Switzerland; 2 Department of Nursing, Bern University Hospital (Insel Gruppe), Freiburgstrasse 38, Bern, 3010 Switzerland; 3 School of Health Professions, Competence Centre Participatory Health Care, Bern University of Applied Sciences, Murtenstrasse 10, Bern, 3008 Switzerland; 4 Academic Practice Partnership of Bern University of Applied Sciences and University Hospital Bern (Insel Gruppe), Murtenstrasse 10, Bern, 3008 Switzerland

**Keywords:** Hospital, Restraint, Patient participation, Pilot projects, Quality improvement

## Abstract

**Background:**

For general hospital settings, effective restraint reduction strategies are lacking. Patient involvement is proven to be useful in restraint reduction in mental healthcare and in long-term care settings. Since such an approach has never been investigated in a general hospital setting, we investigated whether and how patient involvement regarding restraint reduction is feasible in such a setting.

**Methods:**

A pilot study following a participatory action research design was conducted. Qualitative and quantitative approaches were applied to develop and pilot an intervention to reduce restraint by preventive involvement of patients (aged 65+) in Switzerland. The intervention entailed reflecting on the potential risk of restraint use together with the patient within 24 h of admission and jointly defining possible prevention measures. The intervention was piloted for one month on one ward. Data collection for the qualitative evaluation included interviews with eight patients, five nurses, two ward managers and one clinical nurse specialist. These data were analysed by means of content analysis. Data collection for the quantitative evaluation consisted of a survey of nurses and an extraction of data from the electronic patient files. These data were descriptively analysed.

**Results:**

The evaluation comprised the files of 177 patients (pre to post pilot). It was found that that prevalence of restraint was lower during the pilot phase than before (4.8% vs. 10.2%), although a similar number of patients were found to be at a potential risk of restraint use (51.6% vs. 53.3%). In addition, considerably more patients with a potential restraint risk had restraint prevention measures documented (53.1% vs. 10.2%). From the perspective of the nursing staff, feasibility and acceptability of the intervention was not provided. The intervention was considered to be too time-consuming and the target group too unspecific.

**Conclusions:**

The proactive and structured involvement of patients (aged 65+) in the prevention of restraint use might be an approach to reduce restraint use in a general hospital. Patients were positive about being addressed on the topic during the nursing admission interview. However, the effort was regarded to be high. Limiting the intervention to electively admitted patients should be considered to lower the burden.

**Supplementary Information:**

The online version contains supplementary material available at 10.1186/s12877-025-06015-3.

## Background

It is undisputed internationally and across different health care settings that restraints should be used only as a last resort and kept to a minimum, if not abolished altogether [1–3]. According to the definition of the Swiss Academy of Medical Sciences, a restraint is defined as a ‘restriction of freedom of movement and the curtailment of other fundamental rights’ [4]. Restraints can cause serious physical and psychological injuries, such as hematoma, nerve injuries, pressure ulcers or post-traumatic stress disorder, thus prolonging hospitalisation and increasing mortality and cost [5–10]. Therefore, various interventions and strategies have been implemented primarily in psychiatric and long-term care settings to reduce restraint use [11, 12]. For acute somatic hospital settings (hereafter described as ‘general hospital’), lessened activities and few studies have addressed the reduction of restraints, although they are frequently used. Restraint prevalences up to 100% are reported [5, 13–15]. The data on restraint use in general hospitals are very heterogeneous due to different definitions of restraint and also the subpopulations studied (among other things). Previous reduction initiatives in general hospital settings have tended to focus on staff education and multicomponent/complex interventions. The results regarding the effect of such interventions have been very heterogeneous (often little to no effect), and most of these studies have been criticised for methodological weaknesses [16].

In general hospital settings, older and cognitively impaired patients are at greater risk of the use of restraints [15]. Nurses are the key decision makers for restraint use [17–21]. However, it is widely discussed that nurses (and other healthcare professionals) often do not have sufficient knowledge and expertise on restraint use [17–20]. As a result, restraints are often applied in situations where they are not appropriate, for example, to prevent falls, for which the benefits have not yet been proven, while the negative consequences are known [22]. In addition, decision-making and restraint management are influenced by individual attitudes. Whether and when a restraint is initiated is at the discretion of the individual nurse, and it differs considerably among different persons [21]. Since restraint use is an established practice, hardly any ethically and legally required consideration of alternatives and/or preventive measures occurs prior to restraint use [15, 20]. However, there is also a lack of high-quality studies demonstrating effective preventive measures or alternatives to restraint use in general hospitals [16].

In mental healthcare settings, shared decision-making interventions are described as effective in reducing involuntary admission [23–25]. To reduce restraint use in general, it is also recommended to involve patients in the decision-making process, both in mental healthcare (e.g., in the form of advance directives; 26, 27) and in long-term care settings [28]. The preventive measures that could be taken or the restraint type that is preferred if restraints are inevitable is ideally defined with the patient prior to a potential situation leading to restraint use. Since decision making means the weighing up of different options [29], using such an approach makes alternative and preventive measures available as options in the decision-making process. Thus, such an approach might be useful in a general hospital setting as preventive and alternative measures are often not well known by nursing staff and, therefore, are given insufficient consideration. To our knowledge, such an approach has never been investigated in a general hospital setting.

Therefore, we investigated whether patient involvement for the purpose of restraint reduction is feasible in a general hospital and, if so, how involvement can be achieved. For this purpose, we conducted a pilot study to develop and pilot an intervention for preventive patient (aged 65+) involvement to reduce restraint use.

## Methods

### Design

A participatory action research design was used to develop and pilot the intervention [30, 31]. Healthcare professionals working in clinical practice as well as patient representatives were involved in the entire study. This allowed the intervention to be developed according to the needs of the users (patients and nurses) and the practical circumstances. Qualitative and quantitative approaches were applied.

The pilot study was conducted between February 2023 and April 2024 and was conceptualised in three phases in line with Sidani and Braden [32]: development of the intervention and implementation plan (February–August 2023), implementation (September–November 2023) and evaluation (October 2023–April 2024). The project team consisted of researchers from the nursing and health sciences, a Clinical Nursing Specialist (CNS) from the participating hospital group and a patient representative. In addition, an advisory board of three international experts in the use of restraints (mental health care, long-term care and general hospital care) and a national expert in medical ethics provided advice during each phase.

### Setting and sample

The intervention was developed and piloted in collaboration with a ward located in a rural general hospital belonging to a Swiss hospital group. The ward runs 27 beds, which are occupied by both medical and surgical patients. The CNS of this ward became the local coordinator for the study. The hospital group has an up-to-date guideline on restraint use, on which all employees had been trained no more than one year prior to the start of the study. According to the guideline, restraint shall only be used when there are no other means of addressing the reasons leading to their use. For Switzerland, restraint prevalences of 10.2% are reported [33].

The intervention was piloted with patients 65 years or older who gave their consent to participate. The nurses of the ward recruited the patients, and they received written information about the study on admission and had the possibility to clarify questions with the nurse responsible for them. For patients who were cognitively unable to understand the information or give their consent, their legal representative was approached. Written information for patients was shared with two representatives from the Patient Council and optimised based on their feedback.

For the qualitative evaluation, eight patients, five nurses, two ward managers and one CNS were recruited. Patients were recruited by the ward manager or her deputy. She provided the patients who took part in the intervention with written information about the evaluation and enquired about their willingness to talk about their experiences with someone from the project team. The project team was then notified accordingly. Ward managers volunteered to be part of the evaluation and also scheduled nurses from the team for the focus group interview. For the quantitative evaluation, the ward’s CNS provided a survey to the nursing staff and managers. In addition, relevant clinical information from the patient files was obtained from the hospital group’s data centre. The anonymised data covered all patients who were hospitalised on the pilot ward between August 2023 and November 2023, were 65 years or older and had signed a general consent form for further use of their data.

### Intervention and implementation

Information on the development of the intervention and its implementation plan can be found in the supplementary material.

#### Intervention

The intervention was conceptualised as follows (see also Figure S1 in the supplementary material): The intervention will be carried out within the first 24 h after admission to the ward. It could be combined with the nursing admission interview, but the intervention could also be independently administered. Information about a patient’s risk of delirium, falling, cognitive impairment or other common reason for restraint use has to be known in advance. The information could be directly gathered prior to the intervention or learned from the patient file. The intervention will include the following points:

- Based on the findings on the patient’s risk regarding the most common reasons for restraint use, the nurse and the patient reflect on the potential risk of restraints being used during hospitalisation.
- Regardless of the risk, measures aimed at reducing the potential risk of restraint use will then be discussed with the patient. Ideally, the patient will already be aware of methods that give them security and guidance. Otherwise, nurses can address six areas that, according to hospital restraint guidelines, help prevent the most common risks for restraint use: aids, involvement of relatives, orientation/structure, distraction/occupation, urination and defecation and non-pharmacological pain management. The findings (patient’s potential risk of restraint use and preventive or alternative measures to restraints) will be recorded in the patient file to ensure that all team members have been informed of the potential risk and of the preventive measures that should be used before restraint is used. This is intended to increase the likelihood that preventive measures will be exhausted before restraints are used or will be used instead of restraints as required by ethical and legal standards as well as the hospital guideline.

The intervention was implemented on the pilot ward for one month (16 October 2023 to 16 November 2023).

### Data collection

The evaluation focused on feasibility and acceptability was conceptualised according to Sidani and Braden [32]. In terms of feasibility, this comprised the thematic clusters of Material Resources, Contextual Features, Human Resources and Intervention Implementation. Acceptability was assessed using the thematic clusters of Appropriateness, Effectiveness, Risks and Convenience.

Data collection for the qualitative evaluation was undertaken using one-to-one interviews (patients) and focus group interviews (nursing staff, ward managers, CNS). Based on the theoretical model of Sidani and Braden [32], a semi-structured topic guide was developed for each target group by two project team members. The three topic guides covered the same topics in terms of content but were adapted to the perspective of the target groups (see supplementary material). One open-ended main question was formulated for each thematic cluster, supplemented with follow-up and in-depth questions. Pretests with participants from the respective target group were conducted using all of the interview guides for content, comprehensibility and duration. The individual interviews were conducted by one member and the focus group interviews by two members of the project team. All interviews were audio recorded and then transcribed and pseudonymised using MAXQDA software [34]. Once the transcription was complete, the audio files were deleted. Additional notes were taken during the interviews and included in the data analysis in the form of memos. During the focus group interviews, we collected data from nursing staff on their position in the team or additional responsibilities as well as the number of years of professional experience in nursing. Age, gender and the time the intervention occurred during hospitalisation were recorded for the patients.

Data for the quantitative evaluation was collected by means of a survey of nurses, ward managers and CNS and by extracting data from the patient files. A theory-based questionnaire developed by Sekhon, Cartwright and Francis [35] was used for the survey. The questionnaire is based on the Theoretical Framework of Acceptability (TFA) and contains seven items on seven different areas of acceptability (affective attitude, burden, ethicality, perceived effectiveness, intervention coherence, self-efficacy and opportunity costs) and one item about general acceptability. Each item is rated on a Likert scale from 1 (low acceptability) to 5 (high acceptability). Within this theory, feasibility is recognised as a component of acceptability. The questionnaire was translated into the local language by the project team using the forward and backward translation method [36, 37]. Three additional questions on staff characteristics were added to the questionnaire (years of professional experience in nursing; whether trained in intervention [by project team, peer, not trained]; and intervention carried out with patients [yes, no]). The data to be extracted from patient files were defined together with the CNS of the pilot ward. The following information was requested: general patient characteristics, patient-related restraint risk factors (common reasons for restraint use), information on restraint use and documentation of the intervention (potential risk for restraint use, individualised prevention measures). The following restraint types needed to be documented in this hospital according to the hospital group’s guideline: electronic monitoring (fall protection devices, sensor mats, alert systems/movement detection, etc.), low nursing bed, fixed table for wheelchair, bed rail, locked windows/doors, one-to-one supervision, safety mitts, any type of belt fixation in bed or (wheel)chair, special blankets that restrict free movement in bed. The documentation of restraint use was standardised by checking a box in the system. Subsequently, the type of restraint had to be specified in an open text field. The hospital group’s data centre then exported the data for the period of August 2023 to November 2023. To review data quality, the CNS of the pilot ward conducted an internal audit during the pilot phase. Twice a week, she randomly checked whether restraints were being applied and, if so, whether they were also documented in the patient file. She recorded her findings in an Excel list.

### Data analysis

Qualitative data from the one-to-one and focus group interviews were analysed by means of content-structuring content analysis according to Kuckartz and Rädiker [38] using MAXQDA software [34]. Main categories were deductively generated based on the theoretical concepts of feasibility and acceptability [32]. Subcategories were inductively derived. Data was initially analysed separately for each target group and then a cross comparison was made in order to reveal similarities. Coding was carried out by one person (IB). A second person (KT) reviewed the coding of three randomly selected patient interviews and the entire coding of the two focus group interviews. There was a high level of consensus. Pre-final and final coding were critically reflected upon with another person (ST) with reference to the original data. Results (final coding) of the focus groups were verbally validated with people from the respective groups (member checking). Results from the patient interviews could not be validated with the patients as the data analysis only took place after they had left the hospital. Establishing contact would have entailed additional data protection requirements. At the end of the interview, a kind of ad hoc validation took place in which a verbal summary of the findings was created that could be supplemented or corrected by the patients.

Quantitative data was descriptively analysed (numbers, percentages, mean, median). The questionnaire on acceptability was analysed as recommended by Sekhon, Cartwright and Francis [35]. The results per item were analysed (number and percentage per Likert level) and the mean value across the seven items was calculated. Data from the patient files were analysed in total as well as subdivided into pre-pilot phase (August 2023 − 15.10.2023), during pilot phase (16.10–16.11.2023), during pilot phase exclusively with patients participating in the intervention (16.10–16.11.2023) and after pilot phase (17.11.2023–30.11.2023). The proportion of patients for whom at least one restraint was documented, the proportion of patients identified at a potential risk of restraint use, and the proportion of patients identified at a potential risk of restraint and for whom preventive measures were documented, were calculated. As the documentation did not provide for an explicit restraint risk assessment, a possible restraint risk was identified based on the medical diagnoses that implied a risk of falling or a change in cognition or were associated with delirium. In addition, the recorded assessments, such as the Glasgow coma scale for assessing orientation, the Delirium Observation Scale (DOS) or the Stratify falls assessment, were used. Falls, delirium or suspected dementia documented in the nursing admission history were also taken into account. Whether preventive measures were applied was identified by checking the patient file for the mention of, for example, anti-slip-socks or environmental adaptations. SPSS Version 28.0 [39] and Microsoft Excel were used for the quantitative data analysis.

Results of the evaluation were reflected upon with the representatives of the Patient Council, the nurses, the CNS, and the managers of the pilot ward as well as the Advisory Board.

### Ethics

As the intervention was a supplementary approach to implementing the hospital group’s restraint guideline, the study was considered a quality development project, and the responsible ethics committee categorised it as not falling under the Swiss Human Research Act (Req-2023-00412). All participants received written information adapted to the respective phase and gave their consent to voluntary participation.

## Results

### Findings from qualitative evaluation

The eight patients (four male, four female) included in the one-to-one interviews were on average 76.6 years old. With six patients, the intervention was carried out within 24 h of arrival, with one patient, on the third day after admission and with another patient, on the ninth day. The interviews took place one day after the pilot intervention and lasted an average of 23.5 min. Four patients reported experiences of restraint use with their relatives, one person reported having own experiences with restraints. These experiences were described both positively and negatively. The majority of patients considered restraint to be necessary for safety in certain situations.

Five nurses took part in a focus group that lasted 75 min (FG_nurses_P1-5). Four participants had more than five years of professional experience in nursing and three participants had additional responsibilities in professional development on the ward (e.g., implementation of guidelines). Two ward managers and one CNS participated in another focus group that lasted 90 min (FG_management_CNS_P1-3). The participants had an average of 28 years of professional experience in nursing.

Table 1 summarises the main findings of the qualitative evaluation of feasibility and acceptability of the intervention. Each topic is then described in detail.

**Table 1:** Feasibility and acceptability, summary of main findings

Main categories	Subcategories	Patient	Nurses	Management/CNS
**Feasibility**				
Material Resources	Necessary documents were available	x	x	x
Contextual Features	Need for adaptation of the timing to carry out the intervention (within 24 h)	x	x	x
	Importance of a quiet and private location to carry out the intervention	x		
	Nursing admission interview was conducted in a shortened form, which made implementation of the intervention more difficult and time consuming		x	x
	Implementation of the guidelines on falls, delirium and restraint still in progress			x
	Team culture of talking about restraints, which supported implementation of the intervention			x
Human Resources	Nurses were the appropriate staff members to carry out the intervention	x		
	Conflict of resources with other projects		x	x
	Unsuccessful training strategy		x	x
	Co-design: appreciated but challenging		x	x
Intervention Implementation	The intervention was mostly comprehensible	x		
	Uncertain and incomplete implementation in daily practice	x	x	x
**Acceptability**				
Appropriateness	Divergent views on the appropriateness of patient involvement	x		
	Raising awareness of restraint in society was considered useful		x	
	Target group too unspecific		x	x
Effectiveness	Indirect effect by raising awareness among patients and nurses for falls and restraint prevention	x	x	x
	Safety culture		x	x
Risks	No risks	x	x	x
	Hypothetical risks: Disruption of the patient–nurse relationship and moral conflict for nurses		x	
Convenience	Pleasant but unexpected conversation	x		
	Increase in effort due to follow-up tasks		x	x

#### Feasibility

##### Material resources

As reported in the interviews, the information leaflets for patients and the written instructions for nurses were available as intended. Nurses commented that the font on the leaflet for patients was rather small, and the content was too difficult for some patients to understand.

##### Contextual features

The two focus groups revealed that the timing of the intervention was not appropriate. Emergency admissions and incompatibilities with the surgery programme were mentioned as reasons the intervention can rarely be completed within 24 h after admission. According to the participants, it is also difficult to carry out the intervention postoperatively as the patient’s state of health often does not allow for it. Patients mentioned that other issues may be more pressing in the event of an emergency admission and that intervention within 24 h of admission makes little sense in such situations. However, outside of emergency situations, the participating patients felt that the timing of the intervention within 24 h of admission was appropriate. In the focus group interview with the nurses, it was suggested that in this context, the intervention should only be conducted for admitted elective patients or that it should be possible to adapt the timeframe to the individual patient’s situation.

For most patients, it was important that the intervention could take place in a quiet and private place. ‘[…] That there aren’t four other people listening now. I have to say, that’s an advantage’ (patient 3).

In both focus groups, the shortened form of the nursing admission interview was identified as a contextual factor hindering implementation; in addition, the focus group with ward managers and the CNS identified the status of implementation of the hospital’s internal guidelines on falls, delirium and restraint as a contextual factor. It had been decided within the team to implement a shortened form of the nursing admission interview, whereby the actual implementation was handled very differently. These shorted forms of nursing admission interviews meant that certain information was not available as originally intended. ‘Then you just ask casually [during other activities] what their social life looks like [instead of asking in-depth questions in a designated conversation], […] and that’s it’ (FG_nurses_P4). The hospital’s internal guidelines on falls, delirium and restraint are still being implemented. As a result, certain knowledge that would have been helpful for the implementation of the intervention appeared to be missing.

In the focus group with ward managers and the CNS, however, it was emphasised as a beneficial contextual factor that a team culture on discussing restraint use already exists. The nurses support each other in identifying and implementing alternative measures to restraints.

##### Human resources

Patients perceived the nurses as competent to carry out the pilot intervention. From their point of view, the nurses are involved in the processes surrounding restraint and have access to the treatment information. In general, data analysis showed that all patients had a positive view of the nurses and appreciated their work and contact with them.

In both focus groups, scarce time resources and projects taking place at the same time were mentioned as inhibiting factors for intervention implementation. As a solution to this capacity conflict, it was suggested that the intervention be exclusively conducted by designated persons from the nursing team or from the project team. In both focus groups, it was mentioned that the ward manager and the CNS supported the nursing team by sporadically taking over the intervention. However, there was no coaching of the nurses through the CNS in carrying out the intervention.

Difficulties with the intervention training were discussed in both focus groups. While the focus group with the nurses mentioned that many nurses did not attend initial training, which caused confusion about their tasks and resentment towards the intervention, the focus group with the ward managers and the CNS critically reflected that they had underestimated the need for training, which led to too few nurses scheduled for the training sessions. ‘I really underestimated that a little, too. Probably. I thought [the nursing team] would be able to do [the intervention]. [That] it’s not such a big deal. And it really was more complex’ (FG_management_CNS_P2). In addition, both focus groups reported a lack of knowledge about restraint prevention. The nurses stated that they were unsure about the aim of the intervention and did not understand the instructions (e.g., why knowledge about falls at home was relevant in connection with restraint prevention). For the ward managers and the CNS, their lack of knowledge was an obstacle to supporting the nursing team in terms of coaching. In both focus groups, bedside coaching was identified as an appropriate option to address uncertainties. One nurse said: ‘I would have felt very supported, for example, if […] someone had been on site and an emergency or regular admission had been done together, and then it [the intervention] could have been done on that example’ (FG_nurses_P1). None of the nurses who took part in the focus group were aware of the train-the-trainer concept as part of the training strategy. However, it was critically viewed.

Both focus groups contained people who were involved in the development of the intervention as part of the co-design concept. They appreciated being involved as they were able to contribute their views and help adapt the intervention to the setting. Having a dedicated contact person in the project team who they could directly approach with questions was appreciated. However, difficulties with the co-design concept emerged in the focus group with the ward managers and the CNS. First, the aim of the study remained unclear for the participants: ‘Throughout the entire project, you know, even during this preliminary phase, I was always wondering why this [restraints] was the focus. For me, what’s MORE important is what happens before [restraints need to be used], when someone is delirious or at risk of falling. What do I do then to prevent restraint? […] And that’s what happens first, and I have to intervene […]’ (FG_management_CNS_P1). This led to uncertainty in the project team as who was the right person on the pilot ward to collaborate on development of the intervention. The clarification of roles was considered important. Second, the timing of intervention implementation was perceived as a challenge. The time intervals between meetings made it difficult to keep track of the course of the study and the work that needed to be done. Certain organisational tasks that had to be completed by the ward managers and the CNS were also perceived as complicated and time-consuming as different people were often involved. Scheduling the nurses for the training sessions and the systematic recording of the declaration of consent were specifically mentioned.

##### Intervention implementation

The majority of patients found the information leaflets and the intervention easy to understand. Patients mentioned that no additional information was necessary, although some patients expressed difficulty in understanding the term ‘restraint’. It was not clear to them what was meant by a restraint or what types exist. In some cases, it was difficult for the patients to associate restraint with their own situation or with their own images of restraint: ‘[…] I just can’t picture it [restraint]. Yes, in my head, when I think of someone who is really helpless, but it’s not the same [in my situation]’ (patient 9).

Both focus groups showed that there was insecurity regarding the correct implementation of the intervention. ‘That made me so insecure. When you got there, and then after you carried it out, well, has that now been ok or not? Is that what it’s all about? […]’ (FG_nurses_P2). According to the focus group participants, it was particularly difficult to address risks such as delirium or the risk of falls and to define preventive measures. In the focus group of ward managers and CNS, it was also discussed that the documentation of the intervention was perceived as incomplete: ‘[…] I already realised when checking the documentation for completeness that when the patient had a risk factor, they simply marked yes or no, but then didn’t write what kind of risk factor […]. The new stuff, the things that were really new, were not documented […]’ (FG_management_CNS_P1). According to the ward managers and CNS, the instructions on the flowchart and the examples of preventative measures were too complex and difficult, so it was hardly used. The findings from the two focus groups were confirmed by the findings from the patient interviews: According to the patients, the prevention of restraint was not discussed with half of them.

Patients rated the time required for the interviews as adequate, while an increased time requirement was reported in both focus groups. This was attributed to patient difficulties in understanding the intervention, which required additional explanations, and to poorly focussed answers from the patients.

#### Acceptability

##### Appropriateness

Patients had different views on whether it was appropriate to involve patients in the decision-making process. Some patients were in favour, while others clearly felt that as healthcare professionals were responsible, their decisions should be trusted. ‘When you go to hospital or to the general practitioner, you want to get healthier. That’s why it’s logical that you do what THEY say […]’ (patient 7).

The focus group of nurses felt that restraint ought to be addressed more at a societal level, so that it is not just associated with psychiatry. This would increase the possibility of addressing the topic in the general hospital setting. In both focus groups, the target group was described as too unspecific, which resulted in a poorer assessment of appropriateness. In particular, according to the participants, it is hardly possible to implement the intervention for patients with delirium or dementia, who make up a large proportion of the patients on the ward. From the participants’ point of view, the intervention is only appropriate for mobile patients with a risk of falling and for electively admitted patients at risk of post-operative delirium.

##### Effectiveness

It was difficult for the patients interviewed to make a statement about the effectiveness of the intervention. They identified an effect in terms of reflecting on their own situation and assessing prevention options in a more differentiated way. Findings from both focus groups point in a similar direction. An indirect effect on the reduction of restraint was primarily recognised as nurses and patients are sensitised to restraint and fall prevention. As a result, more preventive measures were used. In both focus groups, however, the safety culture in the hospital setting was perceived as an inhibitor to restraint reduction in general. Protection from harm meant that restraints had to be used in certain situations, even when patients reported bad former experiences. In addition, a preventive approach was seen as particularly challenging for patients who were admitted as emergencies and who were confused. It was barely possible to verbally explore preventive measures in these cases.

##### Risks

None of the interviewees (patients or focus groups) experienced any risks during the pilot. Only hypothetical risks were pointed out by the nurses, such as rejecting behaviour from patients or the subject of restraint triggering anxiety in patients that could influence the patient–nurse relationship. However, the intervention was considered to be without risks and some nurses even perceived it as potentially confidence building. ‘[…] when we sit down, the patient has the feeling that someone is REALLY interested in them. So, I don’t see any risk in the conversation [about restraints], I rather see that someone is sitting down and wants to know exactly what could be done. And that can perhaps also be a confidence-building measure […]’ (FG_nurses_P3). However, it was mentioned that divergent assessments between patient and nurse about the necessity of restraints could lead to a moral conflict for nurses. For example, patients might not recognise their risk of falling and the nurse would still have to insert (restrictive) measures.

##### Convenience

The patients described the conversation about restraints with the nurses as pleasant, albeit surprising. For most patients, this was the first time that restraint was discussed during a hospitalisation. ‘Well, it did touch me a little, in a positive sense’ (patient 2). The recommendations on preventive measures were appreciated. In both focus groups, the follow-up tasks were perceived as time consuming, in particular, the documentation required as a result of the intervention and any involvement of relatives’.

### Findings from the quantitative evaluation

A total of 14 out of a possible 32 nursing staff members of the pilot ward completed the survey (participation rate 43.8%). Of these 14 participants, 12 (85.7%) had more than six years of professional experience in nursing. The majority of participants (64.3%) were trained by the project team, and eight participants (57.1%) carried out the intervention in everyday practice (see Table 2).

**Table 2:** Survey sample description (nursing staff)

Participants (*n*)	14
	n (%)
Years of professional experience in nursing	
0–2 years	0 (0.0)
3–5 years	2 (14.3)
6–8 years	3 (21.4)
More than 8 years	9 (64.3
Trained in the intervention by	
Project team	9 (64.3)
Peer	3 (21.4)
No training at all	2 (14.3)
Intervention carried out in everyday practice	
yes	8 (57.1)
no	6 (42.9)

The mean value for acceptability was 2.7, meaning that the participants rated the intervention as neither acceptable nor unacceptable. The individual items of acceptability were assessed very differently, which was also reflected in the assessment of general acceptability (Table 3). Six participants considered the intervention to be either acceptable or unacceptable and the remaining two had no opinion regarding the general acceptability. Participants tended to rate the burden as high, the effectiveness as low and the opportunity costs as high. Participants also felt less confident about carrying out the intervention. Nevertheless, there was a tendency to perceive the intervention as justified and the way the intervention would work as clear.

**Table 3:** Results of the acceptability survey

	1 (low acceptability)	2	3	4	5 (high acceptability)
	n (%)	n (%)	n (%)	n (%)	n (%)
**Affective attitude ** How comfortable did you feel to carry out the intervention?	*Very uncomfortable*	*Uncomfortable*	*No opinion*	*Comfortable*	*Very comfortable*
	1 (7.1)	5 (35.7)	4 (28.6)	4 (28.6)	0 (0.0)
**Burden (recoded)** How much effort did it take to carry out the intervention?	*Huge effort*	*A lot of effort*	*No opinion*	*A little effort*	*No effort at all*
	0 (0.0)	11 (78.6)	2 (14.3)	1 (7.1)	0 (0.0)
**Ethicality ** How fair (justified) is the intervention for patients aged ≥ 65 years in hospitals?	*Very unfair*	*Unfair*	*No opinion*	*Fair*	*Very fair*
	0 (0.0)	5 (35.7)	1 (7.1)	8 (57.1)	0 (0.0)
**Perceived effectiveness ** The intervention has improved (reduced) restraint use.	*Strongly disagree*	*Disagree*	*No opinion*	*Agree*	*Strongly agree*
	4 (28.6)	6 (42.9)	3 (21.4)	1 (7.1)	0 (0.0)
**Intervention coherence ** It is clear to me how the intervention will reduce restraint.	*Strongly disagree*	*Disagree*	*No opinion*	*Agree*	*Strongly agree*
	0 (0.0)	3 (21.4)	3 (21.4)	8 (57.1)	0 (0.0)
**Self-efficacy** How confident do you feel about carrying out the intervention?	*Very unconfident*	*Unconfident*	*No opinion*	*Confident*	*Very confident*
	0 (0.0)	9 (64.3)	1 (7.1)	4 (28.6)	0 (0.0)
**Opportunity costs (recoded)** The intervention interfered with my other priorities.	*Strongly agree*	*Agree*	*No opinion*	*Disagree*	*Strongly disagree*
	1 (7.1)	7 (50.0)	3 (21.4)	3 (21.4)	0 (0.0)
**General acceptability** How acceptable was the intervention to you?	*Completely unacceptable*	*Unacceptable*	*No opinion*	*Acceptable*	*Completely acceptable*
	0 (0.0)	6 (42.9)	2 (14.3)	6 (42.9)	0 (0.0)

In total, 177 patient files could be included in the evaluation (Table 4). Their analysis showed that a total of 23 patients were approached during the pilot phase to take part in the intervention. This equates to 37% of all eligible patients who were hospitalised on the pilot ward during the pilot phase. Of these 23 patients, 12 agreed to participate in the intervention (52%).

The patients were on average 77.2 years old (median 77) and around half were female. The analysis of the patient files indicates that just over half of the patients are at a potential risk of restraint use (indications of frequent reasons for the use of restraint, see chapter data analysis). Among those patients who participated in the intervention, a higher proportion were identified as being at a potential risk of restraint. Otherwise, the patient characteristics for all phases proved to be similar. Before the pilot phase, at least one restraint was documented in 10.9% of patients. During the pilot phase, at least one restraint was documented in 4.8% of the patients and after the pilot phase, in none of the patients. Furthermore, no restraint was applied to any patient who took part in the intervention.

**Table 4:** Patient file analysis – patient characteristics and restraint prevalence

	Total	Before pilot	During pilot	During pilot, only participating patients*	After pilot
Patients (n)	177	92	62	12	23
	n (%)	n (%)	n (%)	n (%)	n (%)
Age (Mean)	77.2	76.5	77.6	77.8	79.3
Age (Median)	77	76	77	77.5	77
Sex (female)	86 (48.6)	40 (43.5)	33 (53.2)	6 (50.0)	13 (56.5)
Patients identified at a potential risk of restraint use	96 (54.2)	49 (53.3)	32 (51.6)	9 (75.0)	15 (65.2)
Patients with restraint use	13 (7.3)	10 (10.9)	3 (4.8)	0 (0.0)	0 (0.0)
*These 12 patients are also included in the ‘During pilot’ column.					

Before the pilot phase, preventive measures were documented in 10.2% of patients with a potential risk of restraint use (Table 5). During the pilot phase, at least one preventive measure was documented in 53.1% of patients at a potential risk of restraint, and for those participating in the intervention, at least one preventive measure was documented in 77.8% of patients at a potential risk of restraint.

**Table 5:** Patient file analysis – restraint prevention

	Total	Before pilot	During pilot	During pilot, only participating patients*	After pilot
Patients identified at a potential risk for restraint use (n)	96	49	32	9	15
	n (%)	n (%)	n (%)	n (%)	n (%)
Preventive measures documented (yes)	24 (25.0)	5 (10.2)	17 (53.1)	7 (77.8)	2 (13.3)
*These 9 patients are also included in the ‘During pilot’ column.					

The following preventive measures were documented as part of the intervention: Wearing suitable shoes for walking, rollator/walker within easy reach, bedside bell easily accessible, calling for assistance when required (for walking or other activities), adequate light when walking and glasses within easy reach. All restraints noted during the audit were identified as recorded in the patient files. Audits took only place during day shifts.

## Discussion

An intervention for the preventive involvement of patients (aged 65+) to reduce restraint use in the general hospital setting was co-designed, piloted for one month on one ward and evaluated from the perspective of nurses, ward managers, CNS and patients. The intervention entailed identification of potential risk for restraint use in all patients (aged 65+) within 24 h of admission to the ward, reflecting on the potential risk with the patient and jointly defining prevention measures. The information on the potential risk and preventive measures was subsequently to be noted in the patient file. The evaluation of the intervention showed that although the topic was considered relevant, feasibility and acceptability was not given from the perspective of the nurses, ward managers and CNS. The intervention was considered too time consuming and the target group too unspecific. Furthermore, the implementation strategy was not successful. The need for training was underestimated, and other projects conducted on the same ward at the same time made implementation more challenging. Patients had a different view of the intervention. The majority of them were positive about the intervention and appreciated the participatory approach. Even tough patients expressed a high level of trust in the healthcare professionals. For some patients, it was clear that the healthcare professionals ought to make the decisions and that these should be followed. The evaluation of the patient files showed that the prevalence of restraint was lower during the pilot phase than before, although a similar number of patients was found to be at a potential risk of restraint use. In addition, considerably more patients with a potential risk for restraint use had restraint prevention measures documented.

Although a co-design approach was used with the involvement of nurses and the CNS of the pilot ward as well as patient representatives, the intervention proved to be challenging to implement in everyday practice. During the development of the intervention and implementation plan, certain prerequisites were not sufficiently clarified, and the roles and opportunities for co-design seemed to have been insufficiently defined. It became apparent during implementation that the nursing admission interview was implemented less systematically than expected. Accordingly, information required for the identification of a potential risk for restraint use was not available as anticipated but had to be established, resulting in the need for extra effort. Similarly, the existing guidelines on delirium, falls and restraint of the hospital group was less consistently implemented. Consequently, an understanding of the interrelationships between these topics and the associated restraint reduction approaches was to some extent lacking. Despite the involvement of the nurses and the CNS of the ward, the discrepancy between the formal requirements and effective implementation in practice remained undetected and the corresponding need for training was underestimated. For example, implementation strategies were proposed in the focus groups that had initially been planned but were subsequently cancelled due to the limited staff resources and the misjudgement of the need for training. This could either indicate that not the appropriate staff members were involved in the development or that the opportunities for co-design were not sufficiently recognised. A top-down approach is still widespread in hospital settings [40, 41]. The pilot ward staff involved in the study may not have been accustomed to being involved in the design of interventions/projects or to having the ability to question and change the fundamental elements of an intervention/project. In a top-down organisation, it may be even more important to reach a common understanding about the involvement of the clinical perspective. Roles, timing, level of involvement and responsibilities should be clarified in a differentiated way and recorded in writing [42]. These aspects might have been insufficiently addressed here.

Based on the interviews with patients, it became apparent that they appreciated being involved in decision-making but had not expected it. Patients expressed great trust in the nurses and considered the decisions made by the nurses to be in the patients’ best interests. If a nurse assessed restraint to be necessary, this assessment was generally trusted. This viewpoint is contrary to the findings in the psychiatric setting [43] in which there has been and is a lot of pressure from patients to reduce coercion and restraint [27, 44]. In the psychiatric setting, active participation has been demanded by patients and has proved to be effective in reducing coercion [23]. Hence, the general needs are likely to differ between settings, and approaches from psychiatry to reduce restraint may not transfer to the general hospital setting, at least not yet. Indeed, the patient group treated on the pilot ward was found to be older and from rural areas. This is likely to be a patient group that is not familiar with being involved in decision making. Yet it can be assumed that the patient group will change in the future with generations that have different expectations and experiences regarding their involvement in decision making [45].

Not only the evaluation from the patients’ point of view but also the evaluation from the nurses’ point of view showed that the involvement of patients in decision making appears to be little institutionalised to date. This was reflected in significant insecurities related to conducting the intervention. It was difficult for nurses to talk to patients about risks and discuss preventive measures. Thus, communication skills seemed to have been insufficiently addressed during implementation. In addition, the burden and the opportunity costs were considered high and the benefit of preventive patient involvement in reducing restraint was considered to be low. The intervention comprised an identification of a potential risk for restraint use on admission. Risk assessment is known to be perceived as complex and time consuming by nurses, especially for older patients [46]. Thus, from the nurses’ point of view, effort and benefit do not seem to be in balance. One of the reasons for the perceived time consumption and imbalance could be that the guidelines and the corresponding risk assessments are divided by topic or risk instead of being linked, which would save time [47]. International guidelines primarily focus on one patient safety risk, and interrelationships between several risks are hardly considered. This also applies to the guidelines of the hospital group on whose ward the pilot took place. As a result, risk assessment is often incomplete and, therefore, prevention possibilities are improvable [46]. Our results confirm the findings insofar as documentation analysis showed that more prevention measures were applied during the pilot phase. Because of the intervention, nurses were required to talk to patients about risks and define preventive measures, which appears to have had the desired effect. The intervention also called for the interlinking of risks, as the reasons for restraint use are usually multifactorial. We expected our intervention to prompt nurses to make this connection, even though to date this has rarely been covered by the available guidelines. The fact that nurses did not make the expected connection could be another reason for the critical assessment of the intervention by the nursing staff, management and CNS. Our trainings did not seem to have made these connections clear enough either.

Finally, the study points to personal beliefs and social norms as barriers for de-implementing restraint use. Both the interviews with patients and the focus groups with nursing staff revealed that a culture of safety is prevalent. Restraint in general hospitals is viewed less critically as it is seen as justified in ‘society’, which is largely in line with the existing evidence [48]. Patients are also only hospitalised for a short time. As the restriction of personal freedom is only of short duration, this may be considered less of a problem. The lack of knowledge of restraint use as low-value care and the associated pervasive personal beliefs and social norms are known barriers in the de-implementation of low-value care [49, 50]. In addition to institutional- and staff-related contextual factors, the knowledge and expectations of patients and relatives are identified as relevant facilitators and barriers in de-implementation [50]. During de-implementation, activities with no or little benefit based on the latest evidence are omitted. Due to personal beliefs or social norms, omitting such activities can cause anxiety among patients that not everything is being done to prevent a fall or similar adverse event; in the case of restraint use, this is especially the case among relatives. On the part of the healthcare professional, failure to fulfil such expectations can, in turn, lead to a fear of accusation if activities are omitted and an adverse event occurs. In connection with restraint use, this is a known barrier to reduction [51–53]. It therefore seems important to actively involve patients and relatives in the decision-making process. However, the results of this study indicate that not only active involvement in the decision-making process is required but also that education of patients and relatives on restraint use as low-value care is necessary. As such, it was also pointed out in the focus group with the nurses that restraint use should be made better known as low-value care at a societal level. Doing so would enable a differentiated ethical assessment of the risks and benefits of restraints by all involved.

### Recommendations

Based on the findings of this study, we summarise that the proposed intervention to reduce restraint may be useful but the patient group should be further specified and the implementation strategy revised. In addition, the effectiveness of the intervention needs to be verified in a larger study with a control group. With regard to the patient group, it should be examined whether in a first step, only electively admitted patients should be included in the intervention. Reflection on the results with representatives from the Patient Council revealed that a lot of information is sent home to patients before they are admitted to the hospital. According to them, it would be conceivable to send information on restraint use prior to hospitalisation, including an invitation to think about measures that will offer patients security and guidance. At the same time, information about the use of restraints as a type of low-value care that should be omitted can be provided, thereby also addressing the expectations and beliefs of patients and relatives. This could have a positive effect on the time needed to carry out the intervention. Such an approach might also allow for randomization of patients to an intervention or control group/ward.

Although the intervention and the implementation plan were developed through co-design and the context of the pilot ward was considered, the implementation strategy was not successful. The implementation plan was not fully put into practice. The e-learning refresher sequences were hardly used, the train-the-trainer concept was not known to the participants and the coaching offered by the project team during the pilot phase was barely utilised despite expressed uncertainties. Responsibilities and monitoring of the execution of the implementation plan must be better clarified. Furthermore, the reflection on the results with the CNS who coordinated the study on site showed that the development and implementation plan should also describe in more detail the involvement of the nursing team in the development and implementation by the CNS. Other methods of involvement within the co-design approach should also be considered (e.g., workshops, simulations), and the roles and competences of the persons involved should be better clarified. In addition, it became apparent that the prevention of restraint use requires knowledge of various topics/phenomena as well as knowledge of the interlinking of these topics/phenomena. The need to explicitly address these links was not recognised during development and should be considered in future interventions on restraint prevention. The state of implementation of existing guidelines and the corresponding knowledge should also be better assessed. The need for implementation measures could then be better tailored.

### Limitations

The intervention was only implemented with 12 patients and in one ward, which should be considered in an interpretation of the results. Moreover, the patients were not randomly included for the intervention. In line with the general practice at the hospital group, it was expected that general consent for further use of patient data would be systematically recorded, but this was only the case at the start of the pilot phase. Therefore, it is possible that the patient data for the period before the start of the pilot phase is not representative. Generalisability of the results is thus limited. The time periods included for the comparison vary due to considerations of practical implementation. In particular, the phase after the pilot measurement is kept very short. Extending this period would have further delayed the availability of the data. This variation must be taken into account when comparing data across phases. Furthermore, a documentation bias could exist as the preventive measures might just have been better documented as part of the intervention. In addition, documentation of restraint use was only audited twice a week during the pilot phase and only during day shifts. Prevalence of restraint use might thus be underestimated. However, this underestimation is likely to affect all phases of this study equally. It should also be noted that pharmacological restraints were not recorded in this study. Finally, the study only included the nursing profession. Other professions (e.g., physicians) are likewise involved in the use of restraints but were not included in this study.

## Conclusion

The proactive and structured involvement of patients (aged 65+) in the prevention of restraint use might be an approach to reducing restraint use. Patients were positive about being addressed on the topic during the nursing admission interview and reflecting on prevention measures with the nurse. However, nursing staff critically assessed the feasibility and acceptability of the intervention. They consider the effort to outweigh the benefit. Nevertheless, the evaluation of the patient files shows that the documentation of preventive measures increased during the pilot phase. However, the findings of this pilot study must be verified in a larger study with a control group. Limiting the intervention to electively admitted patients should thereby be considered. This might also enable randomisation of patients. In addition, the personal beliefs of the nursing staff, patients and relatives as well as social norms should be explicitly addressed during implementation.

## Electronic supplementary material

Below is the link to the electronic supplementary material.

Supplementary Material 1

## Data Availability

The datasets used and/or analysed during the current study are available from the corresponding author on reasonable request.
