# Supplementary material for: Restraint reduction in general hospital care by preventive patient involvement: a pilot study
Source: BMC Geriatr. 2025 May 20;25:358. doi: 10.1186/s12877-025-06015-3 (PMC12090649; doi:10.1186/s12877-025-06015-3)
Supplement: Supplementary file 1 — Supplementary Material 1 [file 12877_2025_6015_MOESM1_ESM.pdf]

Restraint reduction in general hospital care by preventive patient involvement: a pilot study

Supplementary Material

Content

1.	Development of the intervention and implementation plan	1
2.	Implementation	7
3.	Semi-structured topic guides for interviews	7
	3.1 Interview guide patients	8
	3.2 Interview guide focus group nurses	12
	3.3 Interview guide focus group ward management and CNS	16

1. Development of the intervention and implementation plan

The intervention and implementation plan were developed in collaboration with the CNS and five nurses from the pilot ward as well as seven patient representatives from the hospital group's Patient Council and the Advisory Board. The CNS recruited the nurses. Patient representatives were recruited through the office of the council, and it was specified that participants should be aged 65 or older and have hospital experience.

Development of the intervention and the implementation plan took place in a structured and iterative manner. Table S1 summarises the content of the procedural steps. First, the project team synthesised the literature and outlined an approach based on the literature and the distinct expertise of each of the project team members. This initial outline was discussed with the CNS from the pilot ward and was then extended into a first draft of the intervention and the implementation plan. This draft was then presented to the Advisory Board in an online meeting and ad hoc feedback was obtained. In addition, the draft was the focus of structured discussions with representatives of the hospital group's Patient Council and nurses from the pilot ward. Project group members also spent time work shadowing on the ward to ensure that the planned intervention was compatible with the conditions on site. The feedback was then discussed within the project team and with the pilot ward's CNS, and adjustments were

accordingly made to the intervention and the implementation plan. The revised version (second draft) was sent to the Advisory Board for written feedback. The feedback was synthesised by the entire project team, and the intervention and implementation plan were finalised with the ward's CNS. This was followed by two pretests. In one, the medical ethicist of the Advisory Board made himself available to have the intervention tested on him. In the other one, a representative of the Patient Council agreed to watch a simulation of the intervention and provide feedback (the representative himself did not want to take an active role in the simulation). The simulation was performed by a project team member who took on the role of the nurse and a member of the research unit unrelated to the project who took on the role of the patient. The pretest led to minor adjustments to the implementation process, but no adjustments were made to the intervention. It was emphasised that, from an ethical point of view, a clear distinction should be made as to the point at which the 'conversation' is no longer part of the standard treatment and the intervention begins. The patient representatives emphasised that restraint may need to be explained.

Table S1: Summary of the content of the development of the intervention and implementation plan

Development of the intervention and implementation plan		Feedback / Information learned
Outline	Intervention:<br>Restraint use and ways it could be prevented should be discussed during nursing admission interview. Topics to discuss in interview:<br>• Individual risk of patient for restraint use<br>• Exploring measures to prevent restraint use with the patient<br>• Enquiring about preferences if restraint is unavoidable	
	Implementation plan:<br>• Implementation is intended through e-learning.	According to CNS, nurses are overloaded with e-learning.
First draft	Intervention:<br>Restraint use and ways it could be prevented should be discussed following the nursing admission interview. Topics to discuss:<br>• Assessing risk of restraint use together with patient and/or patient's relatives/legal representatives<br>• Defining possible preventive or alternative measures together with patient<br>• Enquiring about patient’s previous experiences with restraints and preferred restraints (if restraints are unavoidable)<br>• Recording of restraint risk and preventive or alternative measures to restraint in the patient file → Increasing the likelihood that preventive measures will be used or at least exhausted before the use of restraints as required by ethical and legal standards as well as the hospital’s guideline.	• Nursing admission interviews are not systematically conducted on the pilot ward.<br>• Asking patients about preferred restraints is not considered realistic since patients have little or no experience with restraint, few recurring patients (difference compared to psychiatric care).
	Implementation plan:<br>On site training of the nursing team in a real or simulated patient situation by the project team, e-learning sequences and pocket cards.	• The transfer of only theoretical knowledge is unlikely to be effective.<br>• From an organisational point of view, it is not possible to train several nurses at the same time.<br>• The fact that the hospital has already implemented a guideline on restraint use/reduction must be better emphasised.<br>• Nurses are overloaded with pocket cards.
Second draft	Intervention:<br>Intervention is carried out within the first 24 hours after admission to the ward. It can be combined with the nursing admission interview, but the intervention can also be conducted independently of the nursing admission interview. The information about patient's risk of delirium, falling, cognitive impairment and other common reasons for restraint use must be known in advance. This information can be directly gathered before the intervention or learned from the patient file. The intervention should include the following points:<br>• Based on the findings on the patient's risk of the common reasons for restraint use, the nurse and the patient reflect on the risk that restraint might be used during hospitalisation.	Largely comprehensible, only suggestions for a clearer description of the inclusion and exclusion criteria.

	• Regardless of the present risk, measures aimed at reducing risk of restraint use are discussed with the patient. Ideally, the patient is already aware of methods that give them security and guidance. Otherwise, nurses can address six areas that, according to hospital restraint guidelines, help prevent the risk of restraint use: aids, involvement of relatives, orientation/structure, distraction/occupation, urination and defecation, non-pharmacological pain management.<br>• The findings (patient's risk for restraint use and preventive or alternative measures to restraint) are recorded in the patient file to ensure that all team members are informed about the risk and preventive measures that should be used before restraint is used. This is intended to increase the likelihood that preventive measures will be used or at least exhausted before the use of restraints as required by ethical and legal standards as well as the hospital's guideline.	
	Implementation plan:<br>• E-learning refresher courses on the guideline on restraint use of the hospital group.<br>• On-site trainings by the project team with a train-the-trainer concept. In this concept, some nurses on the pilot ward are trained in a real or simulated patient situation by the project teams. These nurses then train their co-workers, allowing them to implement the training very flexibly in terms of time.<br>• Nurses trained by the project team as well as the ward's CNS are intended to act as multipliers.	No comments.
Final version	Intervention:<br>No changes are made compared to the second draft.	Restraints might need to be explained when asking patients for participation.<br>Clear distinction between the end of the standard admission interview and the beginning of the intervention.
	Implementation plan:<br>The implementation is carried out in a step-by-step process with sufficient time for training, which also takes holiday absences/annual leaves into account.<br>• Study/project is presented at a team meeting two months before the start of the pilot phase using a poster that remains in the ward as a written memory aid.<br>• Three short (six to eight minutes) e-learning refresher courses on the definition of restraint, patient-related risk factors and restraint prevention measures based on the hospital group's guideline are developed and made available for individual training two months before the start of the pilot phase.<br>• Video sequences of the intervention are made available for individual training and refresher training two months before the start of the pilot phase.<br>• Two on-site trainings in a real or simulated patient situation according to the train-the-trainer concept are scheduled four and two weeks before the start of the pilot phase (Train-the-trainer: Nurses of the pilot ward who take part in the trainings subsequently train their co-workers). Nurses trained by the project team as well as the ward's CNS are intended to act as multipliers. Additionally, a flow chart with the intervention process is provided on laminated sheets (see Figure S1). On the back of this document, the six main areas that can, according to the hospital's guidelines, be addressed to prevent restraints are listed (aids, involvement of relatives, orientation/structure, distraction/occupation, urination and defecation, non-pharmacological pain management; see Figure S2). These flow-charts are designed to be taken into the interviews as a memory aid when conducting the intervention.<br>• Coaching by project team once or twice per week during piloting.	

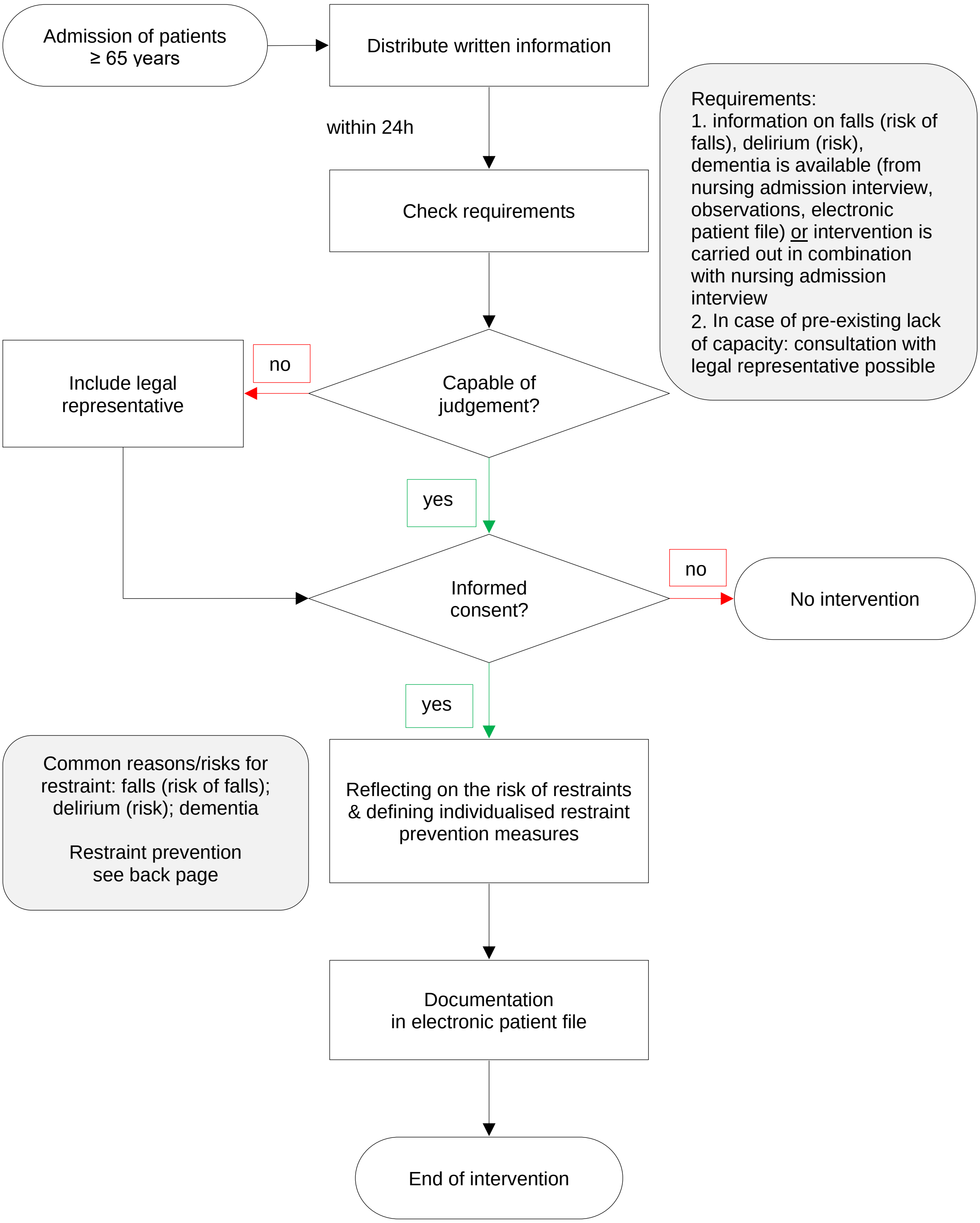

Figure S1: Flowchart Intervention (Front page)

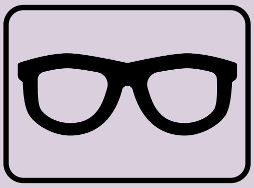

Aids

- Footwear?
- Visual/hearing aids?
- Other aids that could/should be provided?

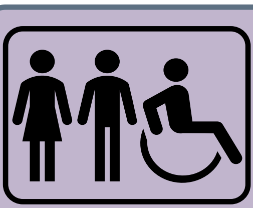

Involvement of relatives

- Available?
- Available if the situation changes?
- If yes, during what timeframes? How can they be contacted?

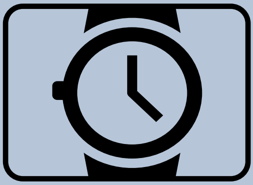

Orientation/structure

- How could orientation be improved? (watch, calendar [from home], sign with location)
- What helps to promote a sense of familiarity? (photos, items, pictures)
- Accustomed to having written reminders? Are they welcome?
- Is there a regular daily routine?
- Other biographical details that could be important for the hospital stay?
- Special blanket or pillow?

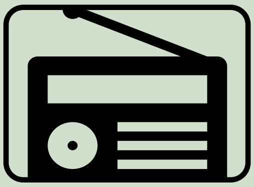

Distraction/occupation

- What do you like to do? What do you do at home that could also be offered in hospital or that could be brought to you from home? (puzzles, audio plays, colouring books, music, TV, etc.)

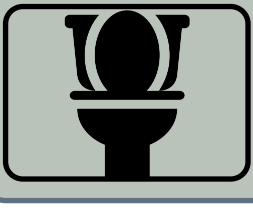

Urination and bowel movements

- Any habits or ailments that could be relevant during a hospital stay? (e.g., going to the toilet every two hours at night)
- Do you need support with fluid intake?

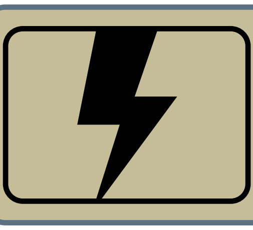

Non-pharmacological pain management

- Non-pharmacological interventions that help at home?

Figure S2: Flowchart Intervention (Back page)

2. Implementation

The intervention was implemented on the pilot ward for one month (16 October 2023 to 16 November 2023). It was planned that all eligible patients would be invited to participate. The implementation was jointly designed with the staff of the pilot ward and was conceptualised as described in table S1. It was necessary during implementation to deviate from the implementation plan for the on-site trainings. More nursing staff than planned took part in the training and were therefore unable to be trained in a real patient situation as planned. Therefore, the interview that was part of the intervention was simulated during the training sequence.

3. Semi-structured topic guides for interviews

The original interview topic guides are in German. The versions integrated here are non-professional translations.

3.1 Interview guide patients

Demographic data:

Age: ……………

Gender: ◻ male ◻ female ◻ diverse

Days since intervention: …………..

Interview guide patients (feasibility and acceptability)

Implementation date of the intervention:…………
Interview location: ◻ Patient room ◻ Room [*name*]
Presence of relatives: ◻ Yes ◻ No
Duration of interview:…………..min.

<table>
<tr><td colspan="3">Conversation start</td></tr>
<tr><td colspan="3">- Express thanks for interest and participation, brief personal introduction
- Reason for conducting the interview: to discuss how the discussion on measures of restraint was perceived as part of the Re-Duct project [intervention].
- The interview will last around 30 minutes and can be paused or terminated at any time.
- If you do not understand a question, please ask. I will be happy to repeat it or rephrase it a little differently.
- The interview is recorded. It is then transcribed without any reference to your person (anonymized).
- Recording is deleted immediately afterwards. Data is evaluated in such a way that it is not possible to trace the person from whom it originated (anonymized).
- Before I start the interview, I obtain the interviewee's consent as to whether it is okay to conduct and record the interview.
- Start recording:
- I ask for the interviewee's consent again to see if it is okay to record the interview.</td></tr>
<tr><td colspan="3">Start of interview</td></tr>
<tr><td colspan="3">- I would like to discuss with you how you have perceived the conversation as part of the Re-Duct project [intervention]. It is about your individual perception.
- You entered the hospital this week and had a conversation with the nurse about restraints [intervention]. I would like to come to the first question:
- Thinking back to that conversation [intervention]..</td></tr>
<tr><td colspan="3">How did you experience the conversation [intervention]?</td></tr>
<tr><td>Questions to keep the interview going</td><td>Additional questions</td><td>Checklist</td></tr>
<tr><td>- Can you tell me</td><td>Alternative question, in case of comprehension difficulties:</td><td>Convenience</td></tr>
</table>

more about it?<br>- What did you mean by that?<br>- Can you give me an example?	- How was it for you to talk about restraints?<br>○ What was rather pleasant/rather unpleasant for you?<br>○ Why did you find it rather pleasant/rather unpleasant?<br>○ Is there anything that we could do different?	*Reaction to conversation?*<br>*How easy was it to start a conversation, to talk about restraints?*<br>*Comprehensibility?*
**How comprehensible and logical was the information provided in advance about the conversation [intervention] and the procedure for you during the conversation [intervention] itself?**		
Questions to keep the interview going	Additional questions	Content-related aspects
- Can you tell me more about it?<br>- What did you mean by that?<br>- Can you give me an example?	*Information about the conversation [intervention]:*<br>- In your opinion, what information was easy to understand/less easy to understand?<br>- What information was more/less important for you?<br>- Is there any additional information you would have liked to have? Which information?<br>*Information during the conversation [intervention]:*<br>- In your opinion, what information was easy to understand/less easy to understand?<br>- What information was important/less important for you?<br>- Is there any additional information you would have liked to have? Which information?<br>*Additional aids:*<br>- What would have supported you in understanding the information?<br>- *If used:* What is your opinion on the picture board with the restraints?<br>*Information about the study:*<br>- How do you rate the written information provided?<br>- What did you find easy/difficult to understand?	Implementation<br>*Clarity, comprehensibility, logical process?*<br>*Is the aim of the conversation clear?*<br>*How comfortable did the patient feel?*<br><br>Material resources<br>*Information material, study information understandable?*<br><br>*Is something missing? If so, what?*
**How important do you think it is to talk about restraints on admission to hospital?**		
Questions to keep the interview going	Additional questions	Content-related aspects
- Can you tell me more about it?<br>- What did you mean by that?<br>- Can you give me an example?	- Why do find it important/not important?<br>- How appropriate do you find this conversation to talk about restraints [intervention]?<br>- What motivated you to take part in the conversation about restraints [intervention]?	Appropriateness<br>*Is it ok to talk about restraints?*<br>*How important do patients find the conversation and the topic of restraints [intervention]?*<br>*Is the conversation [intervention] suitable for addressing health problems (e.g. risk of falling/delirium)?*

<table>
<tr><td colspan="3">What do you think are the risks that should be considered during this conversation [intervention]?</td></tr>
<tr><td>Questions to keep the interview going</td><td>Additional questions</td><td>Content-related aspects</td></tr>
<tr><td>- Can you tell me more about it?
- What did you mean by that?
- Can you give me an example?</td><td>- What needs to be done to reduce this risk?
Alternative question:
- In your opinion, what are possible negative consequences of the conversation [intervention]?
- What needs to be done to reduce these negative consequences?
Specific questions in case of comprehension difficulties:
- Was there anything that was unpleasant for you in connection with the conversation [intervention]? If so, what was unpleasant?
o Why was it unpleasant?
o What could be done against that?
- Did the conversation [intervention] trigger any concerns in you? If so, which ones?</td><td>Risks
Negative impact from conversation [intervention]?
Why?
How could it be reduced?
Does conversation cause anxiety?
Why?</td></tr>
<tr><td colspan="3">How useful do you think the conversation [intervention] is in creating opportunities to reduce restraint?</td></tr>
<tr><td>Questions to keep the interview going</td><td>Additional questions</td><td>Content-related aspects</td></tr>
<tr><td>- Can you tell me more about it?
- What did you mean by that?
- Can you give me an example</td><td>- Why do you find it useful/not useful?
- Would you recommend the conversation [intervention] to other patients? And why?
Alternative question:
- What do you think, should this conversation [intervention] be implemented everywhere in the hospital in the future? And why?</td><td>Effectiveness
Is the conversation [intervention] useful to influence risks/alternatives to restraints?
What is the benefit?</td></tr>
<tr><td colspan="3">How did you experience the time, place and scope of the conversation [intervention]?</td></tr>
<tr><td>Questions to keep the interview going</td><td>Additional questions</td><td>Content-related aspects</td></tr>
<tr><td rowspan="2">- Can you tell me more about it?
- What did you mean by that?
- Can you give me an example</td><td>Timing
- What was it like for you to have the conversation [intervention] when you were admitted (or at another experienced time)?
- What do you find positive/negative about it? Why?
- Would there be a better time for the conversation [intervention]? If so, which one?</td><td rowspan="2">Contextual Features
Room, privacy, time (entry?) ok? State of health complicating conversation?
Relatives present, ok?

Human Resources
Time availability of nurse, perceived competence of nurse?
Feelings during the conversation [intervention]?</td></tr>
<tr><td>Location:
- What was it like for you to have the conversation [intervention] in the patient's room?</td></tr>
</table>

	- What kind of room would you have liked for the conversation [intervention]?<br>- Were there any disturbances or interruptions during the conversation[intervention]?<br>- If so, which ones?	Implementation<br>*Were there interruptions?*<br>*Disturbances?*
	Personnel:<br>- Who was present at the conversation [intervention]? (e.g. relatives, doctor, nurse, etc.).<br>- How suitable did you find it to conduct the conversation [intervention] with a nurse?<br>- What was it like for you to have relatives present during the conversation [intervention]?	
	Duration:<br>- How was the duration of the conversation [intervention]for you?<br>- Why would you have liked more/less time for the conversation [intervention]?	
**Summary of the interview, validation**		
- Thank you very much. Those were all my questions..<br>- I would like to summarize the most important points of our interview so that I am sure that I have understood you correctly.<br>- **Summary of the most important results.** I have understood that…<br>- Did I understand you correctly? Is there anything you would like to add or correct?		
**Conclusion of the interview**		
- We are at the end of the interview.<br>- Is there anything else you would like to add? Is there anything else we should discuss?<br>- If you have any questions or would like to enclose something, you will find my contact details in the study information. If you have any questions about restraints here at the hospital, please contact the hospital's nursing staff.<br>- **Fill in demographic data together** (provide information on this).		
**Thanks and goodbyes**		
- Thank you very much for the interview and your time.<br>- I wish you all the best. Present a small gift.		

3.2 Interview guide focus group nurses

Demographic data participants focus group interview nurses

Years of professional experience in nursing: more than 5 years □ less than 5 years □

Nurse with additional duties/responsibilities at the current position □ Nurse without additional duties/responsibilities at the current position □

Interview guide focus group nursing staff
Date:
Room:
Duration: Min. ……….. Start……. End……..
Moderation:
2nd Moderation:
Material:

Research question, topic	Time	Content
**Welcome and introduction**<br>• Greeting and welcoming everyone.<br>• Thanks to participants for being here today and taking part in the focus group interview.<br>• Informed consent form: Has everyone signed the informed consent form?<br>• Introduce the interviewer by name and function during the interview.	5'	Greeting
**Presentation**<br>• Please introduce yourself (name, function)	5’	Presentation
• We would like to evaluate and optimize Re-Duct [intervention]. This is why we conducted a focus group interview to obtain the views and experiences of nursing staff.<br>• Status of the Re-Duct project [intervention]: pilot phase is now complete, evaluation is underway. Results are expected in the first half of 2024.<br>• Explanation of what a focus group interview is (group discussion, questions prepared, discussion in group important).<br>• It is encouraged that you react to statements made by others and discuss them.<br>• There is no right or wrong, it is all about individual experiences and views.<br>• I guide you through the interview by asking questions.<br>• The interview will last a maximum of 90 minutes.<br>• Interview is recorded on tape. The tape recording is available to the project group members and is deleted after transcription.<br>• The results are used for the project.	5`	Introduction<br>Defining the framework of the interview

• It is important for us to say that the statements made today in the focus group interview are voluntary, will be treated confidentially and the results will not indicate who said what, but only, for example, which interview the quote comes from.<br>• Statements made today should be treated as mutually confidential.<br>• Everyone should have their say and let each other finish. Please do not speak at the same time so that the tape recordings are understandable for the evaluation.<br>• Do you have any questions before the start?<br>• Starting recording devices		
**Guiding questions**<br>We are interested in your experiences in connection with the Re-Duct project [intervention]. I would like to start with the first question:	45`	
**1. Preparation of the project**<br>"How did you experience the preparations for the Re-Duct project [intervention]?"<br>*Topics:*<br>• *Information on the project (e.g. poster, team meeting)*<br>• *Planning the implementation of the project on the ward*<br>• *Exchange with project team on specific topics*		Preparation of the project
**2. Training Re-Duct intervention**<br>"How did you experience the Re-Duct intervention training?"<br>*Topics:*<br>• *Online-trainings*<br>• *Onsite trainings*<br>• *Teach the teacher approach*<br>• *Internal communication/project team (e.g. email about trainings)*<br>• *Confidence in implementation intervention after training*<br>• *Re-Duct intervention (Understanding why and how?)*<br><br>**Additional aids**<br>"What are your experiences with the aids that were provided for the implementation of the Re-Duct intervention?"<br>*Topics:*<br>• *Flyer Re-Duct for patients*<br>• *Flowchart*<br>• *Description of intervention*		Comprehensibility<br>Time required<br>Plannability<br>Training of other employees carried out<br>Confidence to carry out intervention<br><br>Assessment of aids
**3. Carrying out the Re-Duct intervention**		

"What experiences have you had carrying out the Re-Duct intervention in your daily practice?"<br>*Topics:*<br>• *Organisation (e.g. planning conversation with relatives)*<br>• *timing (realistic within 24 hours?)*<br>• *Location*<br>• *Duration*<br>• *Documentation*<br>• *Content added/left out, different relevance of content*<br>• *Confidence (How difficult/easy was it for you)*<br>• *Integration in ward process*<br>• *Experience on-site support from project team*<br>• *Was it possible to ask questions to the project team during the implementation?*<br>• *Training by other members of the care team*<br>• *Staff resources in daily practice / planning in nursing team*<br>• *Internal support options (e.g. CNS, team leaders, other staff members)*<br>• *Experience of patients (reactions?)*		Use in daily practice<br>Comprehensibility<br>Ease of transfer to daily practice<br>Plannability
**4. Assessment of intervention (effectiveness, appropriateness, risks)**<br>"How effective do you think Re-Duct [intervention] is in reducing restraints and why?"<br>"How appropriate do you think it is to talk about restraints for patients aged 65+ on admission?"<br>"In your opinion, what risks should be considered for the patient or have you experienced?"<br>"What risks (consequences) do you see for nurses that could arise from the Re-Duct intervention?"		Effectiveness<br>Appropriateness<br>Risks
**5. Organizational culture, support from CNS and management**<br>"How did you experience the implementation of a project on the subject of restraints in your ward?"<br>*Topics:*<br>• *Topic of restraints present/less present in everyday clinical practice (e.g. in discussions, further training in the team)*<br>• *Relevance of the subject of restraints in everyday clinical practice*		Support through leadership<br>Fit with organizational culture
**6. Evaluation of the project**<br>"How did you experience the evaluation of the project?"<br>*Topics:*<br>• *Questionnaire, interviews*		Evaluation<br><br>Optimization potential
**7. Necessary adjustments**<br>Now, with the opportunity to look back on the pilot phase of the Re-Duct project [intervention]:<br>"What should be done the same or differently in the project?"<br>*Topics:*		Changes due to project

• *Contact persons?*<br>• *Involvement?*<br>• *Information (also type of communication e.g. e-mail)?*<br>• *Type/format of training? Combination?*<br>• *Prior knowledge of restraints?*<br>• *Workload?*<br>• *Documentation?*<br>• *Evaluation, fit/embedding in everyday working life in general/adaptation in this regard (e.g. not realistic within 24h, or only to be expected for patients with hospitalization duration of >3d).*		
**8. Sustainability**<br>“Pilot phase of Re-Duct [intervention] has been completed here in [hospital name], ‘What do you think will happen with the Re-Duct intervention for restraints in [hospital name]?’ 'What will stay, what will go?“		
**Concluding question:**<br>From our side, we have discussed all the issues. Do you think there are any other issues we should discuss? If yes: Gladly, which ones?<br>Thank you for the interesting discussion.	5’	
**Validating the results with the participants**<br>• To increase the reliability that we have understood and processed all statements well, I would like to ask if one or two people would be willing to proofread a tabular overview and comment on it if necessary.<br>• This exchange would take place by e-mail or in a short online session and would probably be at the end of January.	5`	
**Thank you and conclusion**<br>• Thank you very much for your participation and your time.<br>• Please fill in demographic data. Explanations on anonymity.<br>• If you have any questions about this interview, please contact [name] by phone or e-mail.<br>• Presenting a small gift.<br>• Conclusion of the focus group interview.	5`	

3.3 Interview guide focus group ward management and CNS

Demographic data participants focus group management/CNS

Years of professional experience in nursing: ……… years
Role in current position: Leadership with management responsibility at team level ◻ CNS ◻

Interview guide focus group ward management and CNS

Date:
Room:
Duration: Min. ……….. Start……. End……..
Moderation:
2nd Moderation:
Material:

Research question, topic	Time	Content
**Welcome and introduction**<br>• Greeting and welcoming everyone.<br>• Thanks to participants for being here today and taking part in the focus group interview.<br>• Informed consent form: Has everyone signed the informed consent form?<br>• Introduce the interviewer by name and function during the interview.	5'	Greetings
• Aims of the focus group interview: Experiences in connection with the Re-Duct project [intervention].<br>• Status of the Re-Duct project [intervention]: pilot phase is now complete, evaluation is underway. Results are expected in the first half of 2024.<br>• We would like to evaluate and optimize Re-Duct [intervention]. This is why we conducted a focus group interview to obtain the views of management and CNS.<br>• Explaining what a focus group interview is (group discussion, questions prepared, discussion in group important).<br>• It is encouraged that you react to statements made by others and discuss them.<br>• There is no right or wrong, it is all about individual experiences and views.<br>• guide you through the interview by asking questions.<br>• The interview will last a maximum of 90 minutes.<br>• Interview is recorded on tape. The tape recording is available to the project group members and is deleted after transcription.<br>• The results are used for the project.<br>• It is important for us to say that the statements made today in the focus group interview are voluntary, will be treated	5`	Introduction<br>Defining the framework of the interview

confidentially and the results will not indicate who said what, but only, for example, which interview the quote comes from.<br>• Statements made today should be treated as mutually confidential.<br>• Everyone should have their say and let each other finish. Please do not speak at the same time so that the tape recordings are understandable for the evaluation.<br>• Do you have any questions before the start?<br>• Starting recording devices.		
**Guiding questions**<br>We are interested in your experiences in connection with the Re-Duct project [intervention]. I would like to start with the first question:<br>**1. Preparation of the project**<br>"How did you experience the preparations for the Re-Duct project [intervention]?"<br>*Topics:*<br>• *Information on the project (e.g. poster, team meeting)*<br>• *Planning the implementation of the project on the ward*<br>• *Exchange with project team on specific topics*<br><br>**2. Training Re-Duct intervention**<br>"How did you experience the Re-Duct intervention training?"<br>*Topics:*<br>• *Online-trainings*<br>• *Onsite trainings*<br>• *Teach the teacher approach*<br>• *Internal communication/project team (e.g. email about trainings)*<br>• *Confidence in implementation intervention after training*<br>• *Re-Duct intervention (Understanding why and how?)*<br><br>**Additional aids**<br>"What are your experiences with the aids that were provided for the implementation of the Re-Duct intervention?"<br>*Topics:*<br>• *Flyer Re-Duct for patients*<br>• *Flowchart*<br>• *Description of intervention*	45`	Preparation of the project t<br><br>Comprehensibility<br>Time required<br>Plannability<br>Training of other employees carried out<br>Confidence to carry out intervention<br><br>Assessment of aids

**3. Carrying out the Re-Duct intervention**<br>“What experiences have you had carrying out the Re-Duct intervention in your daily practice?”<br>*Topics:*<br>• *Organisation (e.g. planning conversation with relatives)*<br>• *timing (realistic within 24 hours?)*<br>• *Location*<br>• *Duration*<br>• *Documentation*<br>• *Content added/left out, different relevance of content*<br>• *Confidence (How difficult/easy was it for you)*<br>• *Integration in ward process*<br>• *Experience on-site support from project team*<br>• *Was it possible to ask questions to the project team during the implementation?*<br>• *Training by other members of the nursing team*<br>• *Staff resources in daily practice / planning in nursing team*<br>• *Internal support options (use of it?)*<br>• *Reactions of patients and staff*		Use in daily practice<br><br>Comprehensibility<br>Ease of transfer to daily practice<br>Plannability
**4. Assessment of intervention (effectiveness, appropriateness, risks)**<br>“How effective do you think Re-Duct [intervention] is in reducing restraints and why?”<br>“How appropriate do you think it is to talk about restraints for patients aged 65+ on admission?”<br>“In your opinion, what risks should be considered for the patient or have you experienced?”<br>“What risks (consequences) do you see for nurses that could arise from the Re-Duct intervention?”		Effectiveness<br>Appropriateness<br>Risks
**5. Organizational culture**<br>“How did you experience the implementation of a project on the subject of restraints in your ward?”<br>*Topics:*<br>• *Topic of restraints present/less present in everyday clinical practice (e.g. in discussions, further training in the team)*<br>• *Relevance of the subject of restraints in everyday clinical practice*<br>• *Main tasks as management / CNS during project implementation*		Fit with organizational culture
**6. Evaluation of the project [intervention]**<br>“How did you experience the evaluation of the project [intervention]?”		Evaluation

**7. Necessary adjustments**<br>Now, with the opportunity to look back on the pilot phase of the Re-Duct project [intervention]:<br>“What should be done the same or differently in the project?”<br>*Topics:*<br>• *Contact persons?*<br>• *Involvement?*<br>• *Information (also type of communication e.g. e-mail)?*<br>• *Type/format of training? Combination?*<br>• *Prior knowledge of restraints?*<br>• *Workload?*<br>• *Documentation?*<br>• *Evaluation, fit/embedding in everyday working life in general/adaptation in this regard (e.g. not realistic within 24h, or only to be expected for patients with hospitalization duration of >3d).*<br><br>**8. Sustainability**<br>“Pilot phase of Re-Duct [intervention] has been completed here in [hospital name], ‘What do you think will happen with the Re-Duct intervention for restraints in [hospital name]?’ 'What will stay, what will go?“		Optimization possibilities<br><br>Changes due to project
**Concluding question:**<br>From our side, we have discussed all the issues. Do you think there are any other issues we should discuss? If yes: Gladly, which ones?<br>Thank you for the interesting discussion.	5’	
**Validating the results with the participants**<br>• To increase the reliability that we have understood and processed all statements well, I would like to ask if one or two people would be willing to proofread a tabular overview and comment on it if necessary.<br>• This exchange would take place by e-mail or in a short online session and would probably be at the end of January.	5`	
**Thank you and conclusion**<br>• Thank you very much for your participation and your time.<br>• Please fill in demographic data. Explanations on anonymity.<br>• If you have any questions about this interview, please contact [name] by phone or e-mail.<br>• Presenting a small gift.<br>• Conclusion of the focus group interview.	5`	
